# Bioinformatic modelling of SARS-CoV-2 pandemic with a focus on country-specific dynamics

**DOI:** 10.1186/s12889-023-15092-1

**Published:** 2023-01-21

**Authors:** Jakub Liu, Tomasz Suchocki, Joanna Szyda

**Affiliations:** 1 Biostatistics Group, Department of Genetics, Wroclaw University of Environmental and Life Sciences, Kozuchowska 7, 51-631 Wroclaw, Poland; 2 National Research Institute of Animal Production, Krakowska 1, 32-083 Balice, Poland

**Keywords:** COVID-19, Mixture model, Outlier, SIRD

## Abstract

**Background:**

One of the seminal events since 2019 has been the outbreak of the SARS-CoV-2 pandemic. Countries have adopted various policies to deal with it, but they also differ in their socio-geographical characteristics and public health care facilities. Our study aimed to investigate differences between epidemiological parameters across countries.

**Method:**

The analysed data represents SARS-CoV-2 repository provided by the Johns Hopkins University. Separately for each country, we estimated recovery and mortality rates using the SIRD model applied to the first 30, 60, 150, and 300 days of the pandemic. Moreover, a mixture of normal distributions was fitted to the number of confirmed cases and deaths during the first 300 days. The estimates of peaks’ means and variances were used to identify countries with outlying parameters.

**Results:**

For 300 days Belgium, Cyprus, France, the Netherlands, Serbia, and the UK were classified as outliers by all three outlier detection methods. Yemen was classified as an outlier for each of the four considered timeframes, due to high mortality rates. During the first 300 days of the pandemic, the majority of countries underwent three peaks in the number of confirmed cases, except Australia and Kazakhstan with two peaks.

**Conclusions:**

Considering recovery and mortality rates we observed heterogeneity between countries. Liechtenstein was the “positive” outlier with low mortality rates and high recovery rates, at the opposite, Yemen represented a “negative” outlier with high mortality for all four considered periods and low recovery for 30 and 60 days.

## Background

One of the most seminal global events since 2019 has been the outbreak of the SARS-CoV-2 (Severe Acute Respiratory Syndrome – Coronavirus—2) infections in humans, which occurred in December in China [1] and has been following worldwide spread in 2020 (see e.g. World Health Organisation Coronavirus disease situation reports at www.who.int/emergencies/diseases/novel-coronavirus-2019/situationreports). It is commonly believed that the current pandemic has had its roots in the seafood market located in Wuhan [2]. Since late 2019 the virus has spread globally and evolved into several strains throughout 2020 and 2021. The two most common means of infection with the SARS-CoV-2 virus are the so-called respiratory droplets and touching an infected surface. When an infected person sneezes or coughs the viral particles become airborne, can travel up to several meters and infect an oral or nasal cavity of a nearby person [3]. SARS-CoV-2 can survive up to several hours on most surfaces and even several days on certain surfaces [4]. When a person touches an infected surface, it is easy to transfer viruses onto one’s mucus membranes such as eyes, nose, or mouth. The main location of viral replication is lung epithelial cells where the pathogen uses its S protein to bind with the ACE2 receptor of a host cell [5]. After binding, a virus is ready to infect a cell either via direct entry, where the membrane of the host cell and the viral envelope fuse together, or by endocytosis, where an entire virus is engulfed by a part of the cellular membrane [6]. Inside a host cell, a virus uses cellular machinery (mainly the endoplasmic reticulum and the Golgi Apparatus) to assemble new virions. The release of newly produced viruses places a lot of stress on human cells and consequently leads to apoptosis [7] and leads to a major immune response, often referred to as a cytokine storm. This strong inflammation leads to an excess build-up of mucus, mostly in the alveoli. Such a condition is responsible for shortness of breath, which is one of the most common symptoms of the SARS-CoV-2 infection.

Various countries have adopted different policies to deal with the dynamics of this pandemic and, atop those policies, countries also differ in terms of their socio-geographical characteristics (such as climate, total population, population density, etc.) as well as in the public health care facilities. An unpublished study performed by one of the authors has demonstrated a significant impact of the GDP per capita and the Environmental Performance Index on the severity of the pandemic in the given country. Other socio-geographical factors, like population density or median age, also play a role in the development of the pandemic in those countries. Local social habits also severely influence the pandemic dynamics. For example, in many south European countries, social life mainly takes place in public spaces, whereas in Scandinavia people tend to spend more time in smaller groups and closed areas. One of the major factors influencing the severity of the pandemic is the state of the local healthcare system, which can be quantified by the number of hospital beds per a given number of citizens as example. Moreover, it could have been expected that, especially during the initial stage of the pandemic, the severity of country-specific policies will be crucial for its development, but the average daily number of infected cases reported by European countries between February and November 2020 does not correlate with country-specific government stringency indices (Pearson correlation coefficient equal to -0.0230).

Our study aimed to fit SIRD (Susceptible-Infectious-Recovered-Deceased) models to early (30 days), mid- (60 and 150 days), and long-term (300 days) time-frames of SARS-CoV-2 pandemic, separately to each country, to estimate country-specific pandemic parameters. However, the SIRD model only allows for fitting one peak of infection during the pandemic, which in the case of SARS-CoV-2 results in averaging over multiple peaks. Therefore, in addition to SIRD, linear mixture models were fitted to the same set of data to estimate fluctuations in the dynamics of the pandemic. The final goal of the study was the assessment of (dis)similarities between countries applying outlier detection methodology.

## Methods

### Study population

The analysed data comprised cumulative daily numbers of: confirmed cases, deaths, and recoveries, reported for 191 countries, beginning from the 21^st^ of January 2020. This data set represents SARS-CoV-2 Data Repository provided and curated by the Center for Systems Science and Engineering at Johns Hopkins University [8] and was obtained via Github (github.com/CSSEGISandData/COVID-19) using custom-written Python scripts. Separately for each country, the pandemic’s dynamics was modelled for 30 days (D30), 60 days (D60), 150 days (D150), and 300 days (D300), beginning from the first day in which the number of confirmed cases in a given country exceeded zero, what resulted in various calendar dates – depending on a country.

### SIRD model

The SIRD model and the least-squares estimators for its parameters were implemented using custom-written Python scripts following Anastassopoulou et al. [9]. In brief, the model is defined by the following equations:

$$S\left(t\right)=S\left(t-1\right)-\frac{\alpha }{N}S\left(t-1\right)I\left(t-1\right)$$

$$I\left(t\right)=I\left(t-1\right)+\frac{\alpha }{N}S\left(t-1\right)I\left(t-1\right)-\beta I\left(t-1\right)-\gamma I\left(t-1\right)$$

$$R\left(t\right)=R\left(t-1\right)+\beta I\left(t-1\right)$$

$$D\left(t\right)= D\left(t-1\right)+\gamma I\left(t-1\right)$$

where, $$S\left(t\right)$$ represents the number of susceptible individuals expressed by the total country population (N), diminished by the number of infected individuals at time $$t$$$$I\left(t\right)$$, the numbered of recovered individuals at time $$t$$$$R\left(t\right)$$, and the number of dead individuals at time $$t$$$$D\left(t\right)$$, obtained from the Github repository [8]^s^. The estimated model parameters comprise infection rate ($$\alpha )$$ representing the rate of transition from individuals in the susceptible group at time $$t$$ to individuals in the infected group at time $$t+1$$, recovery rate ($$\beta$$) representing the rate of transition from individuals in the infected group at time $$t$$ and to individuals in the recovered group at time $$t+1$$, and mortality rate ($$\gamma$$) denoting the rate of deaths at time $$t+1$$ of individuals representing the infected group at time $$t$$. In our study we estimated country-specific $$\beta$$ and $$\gamma$$, assuming a time unit represented by a calendar day, following the estimators defined by Anastassopoulou et al. [9], given by $$\widehat{\beta }={\left[{\varvec{C}}\Delta {\varvec{I}}{\left(t\right)}^{T}{\varvec{C}}\Delta {\varvec{I}}\left(t\right)\right]}^{-1}{\left[{\varvec{C}}\Delta {\varvec{I}}{\left(t\right)}^{T}{\varvec{C}}\Delta {\varvec{R}}\left(t\right)\right]}$$ and $$\widehat{\gamma }={\left[{\varvec{C}}\Delta {\varvec{I}}{\left(t\right)}^{T}{\varvec{C}}\Delta {\varvec{I}}\left(t\right)\right]}^{-1}{\left[{\varvec{C}}\Delta {\varvec{I}}{\left(t\right)}^{T}{\varvec{C}}\Delta {\varvec{D}}\left(t\right)\right]}$$, separately for the time-frame of 30-days, 60-days and 150-days, and 300-days, counting from the occurrence of the first infected individual in a given country. $${\varvec{C}}\Delta {\varvec{I}}$$, $${\varvec{C}}\Delta {\varvec{R}}$$, and $${\varvec{C}}\Delta {\varvec{D}}$$ represent the cumulative numbers of infected, recovered and dead individuals respectively within the considered time frame.

### Linear mixture model

Linear mixture models were fitted to the daily numbers of confirmed cases as well as to the daily numbers of deaths during the first 300 days of a pandemic using the *GaussianMixture* module of the Scikit-learn [10] Python library, separately for each country and time period. First, models with three normal components were fitted to each country's data reflecting the underlying assumptions of three peaks of the pandemic in this time period. Further on, two-sample t-tests were calculated for the two consecutive means and standard deviations estimated for each country to determine whether this assumption was appropriate i.e. the difference between the estimated means exists. The difference between the means was determined as nonsignificant based on a *P*-value threshold above 0.05 after a multiple testing correction based on the Bonferroni approach, meaning that two, instead of three, components were sufficient for a given country during the first 300 pandemic days.

### Outlier detection

Detection of outlying countries was based on $\widehat{\beta }$ and $\widehat{\gamma }$ from the SIRD models as well as on the estimated means of normal distributions from the linear mixture models. For this purpose, the country-specific data points given by the abovementioned estimates of model parameters (${\varvec{X}}$) were divided into countries located within the estimated decision boundary (non-outliers) and outside the boundary (outliers). Three following approaches implemented via the Scikit-learn were used for classification. (1) Support Vector Machine classifier, implemented through the *svm.OneClassSVM* function, fitting the sigmoid kernel function $f\left({\varvec{X}}\right)=tahn\left(\gamma \left({\varvec{X}},{{\varvec{X}}}^{T}\right)\right)$ and allowing for maximally 20% of countries to be located outside of the estimated decision boundary and thus representing outliers. (2) A classifier based on the Local Outlier Factor, implemented through *neighbors.LocalOutlierFactor* function, classifying countries using their Euclidean distance from the decision boundary, which was estimated based on 20 neighbouring countries. Countries located outside a given distance from this boundary were classified as outlying observations. (3) Density-Based Spatial Clustering approach, implemented through *cluster.DBSCAN* function. In our application, the maximum distance between two samples to form a cluster was set to 0.9, which resulted in forming a single cluster of countries and a set of outlying countries.

## Results

### Epidemiological parameters

**Fig. 1:**
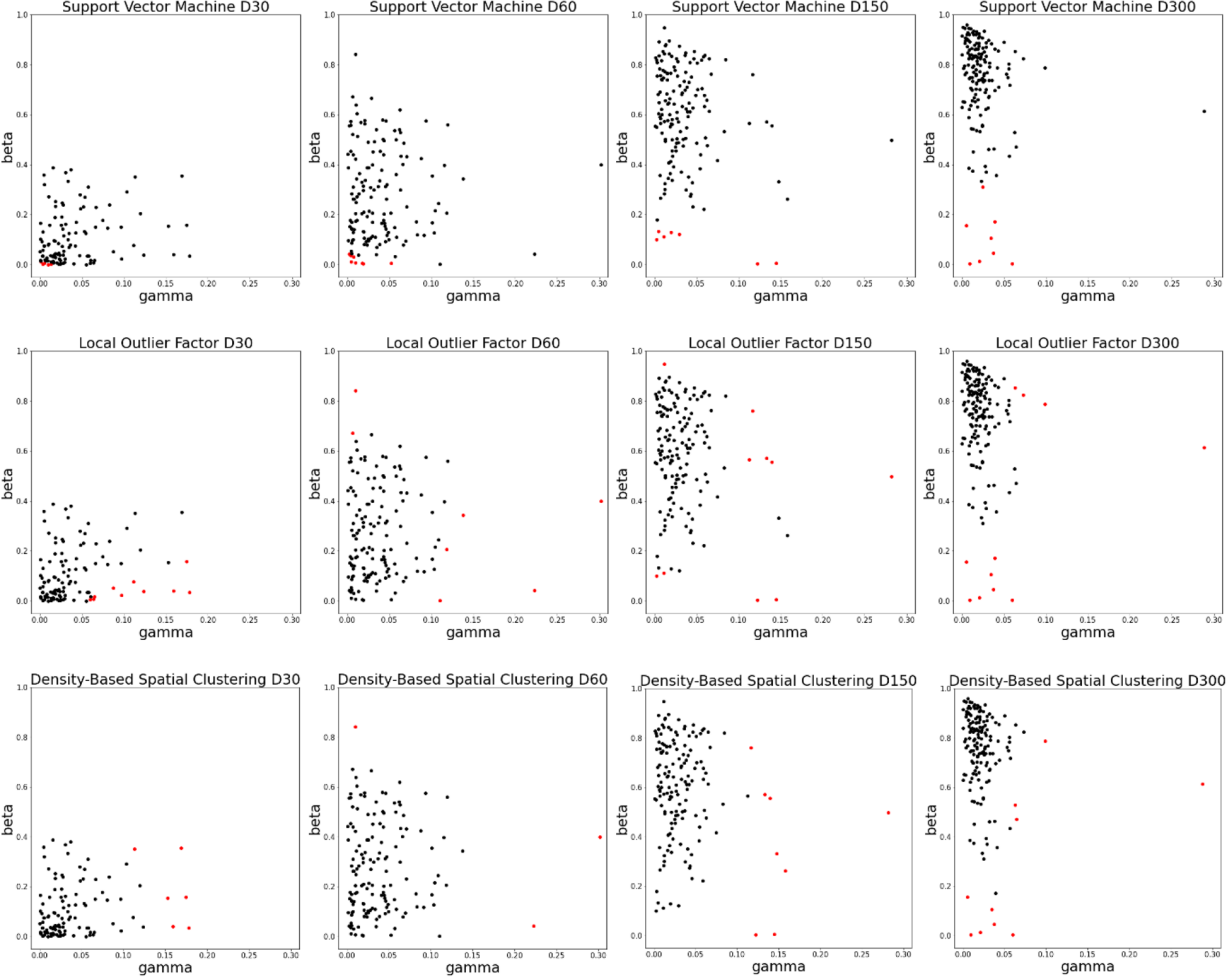
Clustering based on country-specific $\widehat{\beta }$ [individuals] (recovery rate) and $\widehat{\gamma }$ [individuals] (mortality rate) from the SIRD model fitted to the first 30 (D30), 60 (D60), 150 (D150), and 300 (D300) days of the pandemic. Countries classified as outliers are marked in red

Based on recovery and mortality rates estimated from the SIRD model no marked overlap between outliers detected by the three methods as well as between periods (D30, D60, D150, and D300) was observed (Fig. 1 and Table 1). For the longest modelling period of 300 days Belgium, Cyprus, France, the Netherlands, Serbia, and the United Kingdom (UK) were classified as outliers by all three methods. Among them, the UK and the Netherlands, also based on the first 150 days of the pandemic. Still, a pattern emerges in which Yemen was classified as an outlier for each of the considered timeframes, which was due to mortality rates estimated higher than in the bulk of countries amounting to 0.178, 0.223, 0.282, and 0.288 respectively for D30, D60, D150, and D300. During the beginning of the pandemic, those high mortality rates were accompanied by low recovery rates of 0.034 for D30 and 0.041 for D60, which were however much higher when a longer time span was considered. Comparably low recovery rates, albeit with varying mortality rates, starting from the 60^th^ day of the pandemic were estimated for Netherlands (${\widehat{\beta }}_{60}=$ 0.002, ${\widehat{\beta }}_{150}=$ 0.004, ${\widehat{\beta }}_{300}=$ 0.014) and the UK (${\widehat{\beta }}_{60}=$ 0.006, ${\widehat{\beta }}_{150}=$ 0.005, ${\widehat{\beta }}_{300}=$ 0.003), which caused the classification of both countries as outliers.

**Table 1:** mortality rate ($\gamma$) 0–0.30/ recovery rate ($\beta$) and 0–1.0

Country	D30 [$\gamma /\beta$]	D60 [$\gamma /\beta$]	D150 [$\gamma /\beta$]	D300 [$\gamma /\beta$]
Algeria	0.138/0.343			
Austria	0.005/0.006			
Belgium			0.158/0.262	0.037/0.045
Belize	0.123/0.039		0.011/0.113	
Bolivia	0.064/0.008			
Botswana			0.002/0.101	
Brunei	0.007/0.673			
Burundi	0.113/0.352			
Chad				0.063/0.853
Chile	0.001/0.012			
Cyprus				0.006/0.156
Ecuador				0.073/0.825
Equatorial Guinea		0.008/0.030		
Finland		0.005/0.012		
France			0.147/0.332	0.035/0.107
Guinea-Bissau		0.005/0.036		
Guyana	0.153/0.154			
Haiti	0.060/0.006			
Honduras	0.065/0.018		0.030/0.122	
Hungary			0.133/0.571	0.025/0.311
Indonesia	0.088/0.051			
Ireland	0.013/0.003			
Italy			0.140/0.556	0.064/0.472
Liberia	0.112/0.076			
Libya			0.020/0.128	
Liechtenstein	0.010/0.841	0.010/0.841	0.012/0.949	
Lithuania	0.010/0.000			
Maldives		0.003/0.042		
Mauritania	0.169/0.355			
Mexico			0.116/0.762	0.099/0.788
Namibia			0.005/0.133	
Netherlands		0.111/0.002	0.122/0.004	0.021/0.014
Nicaragua	0.302/0.400	0.302/0.400		
Norway	0.004/0.001	0.019/0.004		
San Marino	0.097/0.024			
Serbia				0.010/0.004
South Sudan		0.010/0.007		
Spain			0.113/0.565	0.039/0.172
Sudan	0.175/0.159			0.063/0.530
the United Kingdom		0.052/0.006	0.145/0.005	0.060/0.003
the US		0.018/0.006		
Yemen	0.178/0.034	0.223/0.041	0.282/0.497	0.288/0.615
Zimbabwe	0.159/0.041	0.119/0.207		

Country-specific estimates of $\beta$ [individuals] (recovery rate) and $\gamma$ [individuals] (mortality rate) from the SIRD model fitted to the first 30 (D30), 60 (D60), 150 (D150), and 300 (D300) days of the pandemic, for countries classified as outliers. Outliers classified by one method – white, by two methods – light grey, by three methods – dark grey

**Fig. 2:**
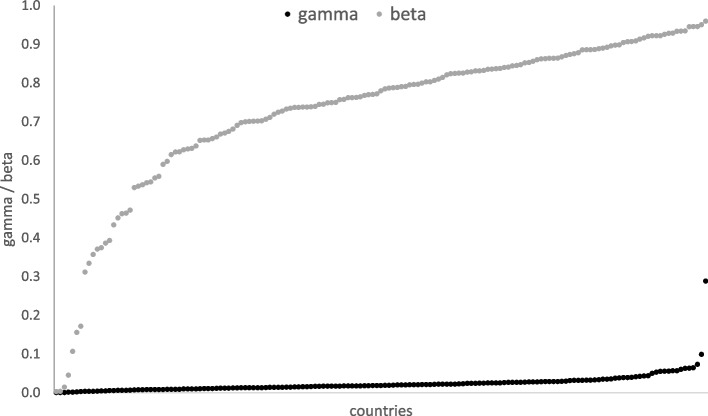
Country-specific $\widehat{\beta }$ [individuals] (recovery rate) and $\widehat{\gamma }$ [individuals] (mortality rate) from the SIRD model fitted to the first 300 (D300) days of the pandemic

**Fig. 3:**
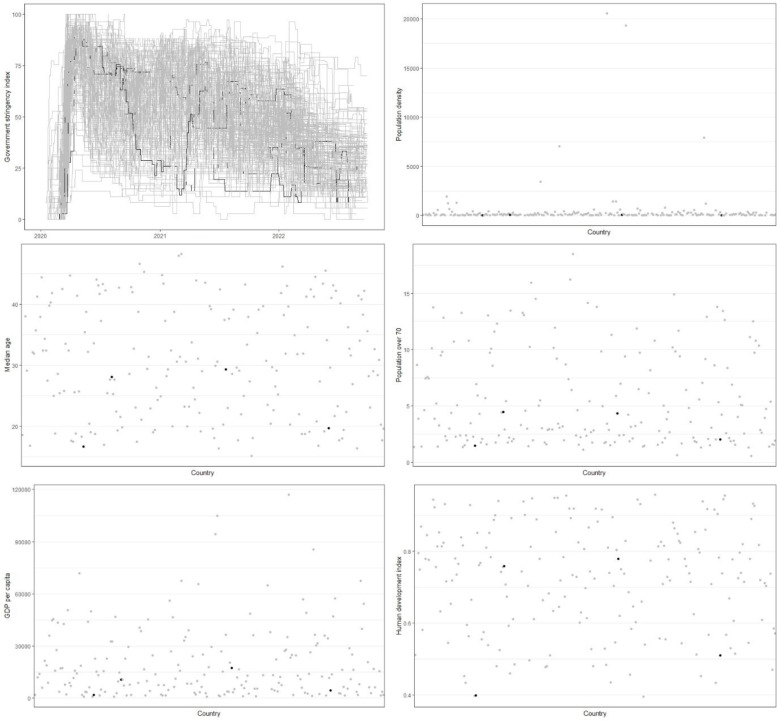
Socio-economic factors of the analysed countries. Countries with a low mortality rate $\widehat{\gamma }$ [individuals]) and a low recovery rate $\widehat{\beta }\, [\mathrm{individuals}]$ (Chad $\widehat{\gamma }=$ 0.063 and $\widehat{\beta }=$ 0.853, Ecuador $\widehat{\gamma }=$ 0.073 and $\widehat{\beta }=$ 0.825, Mexico $\widehat{\gamma }=$ 0.099 and $\widehat{\beta }=$ 0.788), Sudan $\widehat{\gamma }=$ 0.063 and $\widehat{\beta }=$ 0.530), classified as outliers for D300 are marked in black

Considering the longest time span (D300), for each country, mortality rates were lower than recovery rates. Except for Yemen, which was classified as an outlying country, all the remaining countries revealed mortality rates below 10%. On the other hand, estimated recovery rates varied greatly between countries from as low as less than 1% in Serbia and the UK to over 95% in Bahrain, Djibouti, Ghana, Qatar, and Uzbekistan (Fig. 2). Furthermore, for the four countries classified as “positive” outliers at D300 i.e. characterised by a low mortality rate and a high recovery rate (Chad, Ecuador, Mexico, and Sudan), the selected socio-economic factors were visualised comparatively to the remaining countries (Fig. 3).

### Dynamics of the numbers of confirmed cases across time

**Fig. 4:**
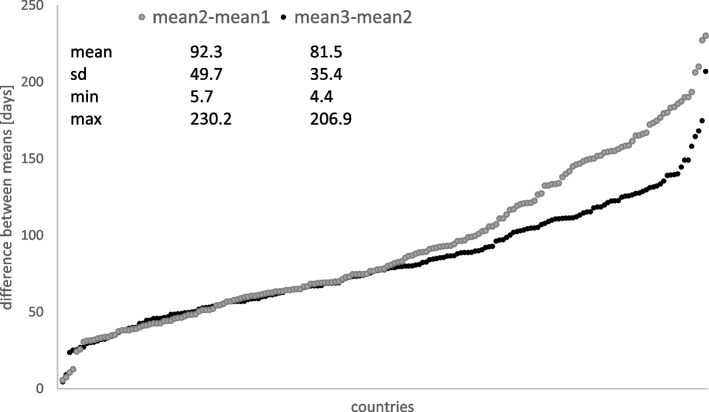
Differences in days between two consecutive peaks of the number of confirmed cases estimated by the linear mixture model for the first 300 days of the pandemic

During the first 300 days of the pandemic, the vast majority of countries underwent three major peaks in the number of confirmed cases (Fig. 4). The two exceptions were Australia with two peaks at day 63 and then the second between the 188^th^ and the 192^nd^ day of the pandemic and Kazakhstan for which the 1^st^ peak was estimated at day 131 and the 2^nd^ peak at day 263. For the remaining countries, the average timeframe between the two first peaks was 92 ± 50 days, with the shortest interval of only 6 days estimated for Suriname and the longest for Liechtenstein – 230 days, followed by the average time between the second and the third peak of 91 ± 35 days varying between 4 days for the Diamond Princess and 207 days for San Marino. Those are four locations with (very) small populations. The earliest peaks were estimated for the Diamond Princess at day 3, 11, and 15 of the pandemic, with small corresponding standard deviations of only 1.7, 2.1, and 7.4 days. Burma “had” its first peak as late as day 205 while Seychelles had the latest 2^nd^ and 3^rd^ peaks at day 263 and 295 respectively.

**Fig. 5:**
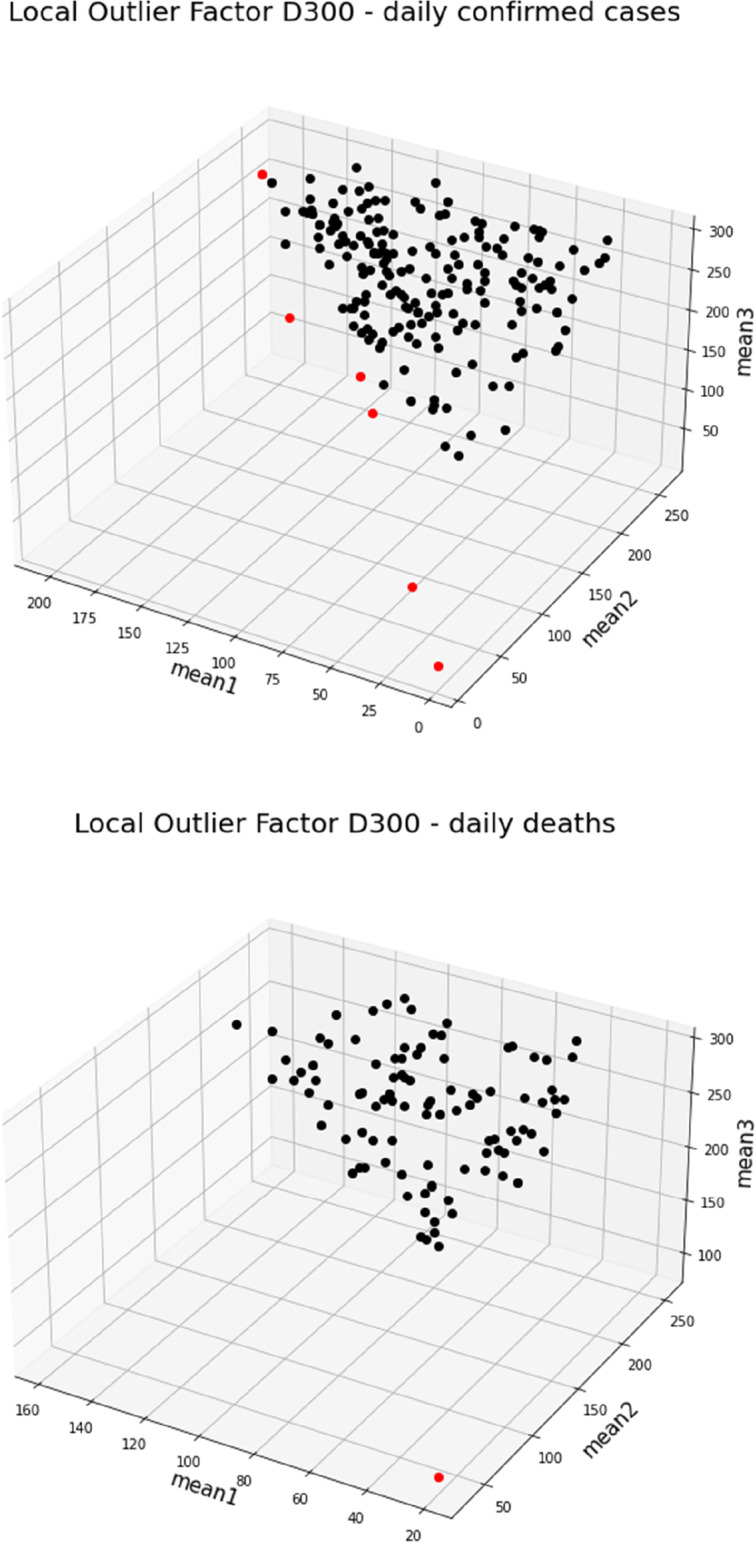
Clustering based on country-specific means of three normal distributions fitted by the linear mixture model to the first 300 (D300) days of the pandemic. Countries classified as outliers are marked in red

The Local Outlier Factor approach identified five countries and the Diamond Princess with outlying patterns of peaks in the number of confirmed cases, which however differ between each other and underlie no clear geographic pattern (Fig. 5). For Burma, relatively late peaks were estimated at days 205, 242, and 271. The opposite was observed for the Diamond Princess (3^rd^, 11^th^, and 15^th^ day) and Tanzania (32^nd^, 45^th^, and 54^th^ day). Quite a similar pattern with all three peaks distributed closely around the middle of the analysed 300-day period was estimated for the Central African Republic (79^th^, 104^th^, and 176^th^ day), Singapore (98^th^, 130^th^, and 184^th^ day), and Gambia (151^st^, 162^nd^, and 194^th^ day).

### Dynamics of the numbers of deaths across time

**Fig. 6:**
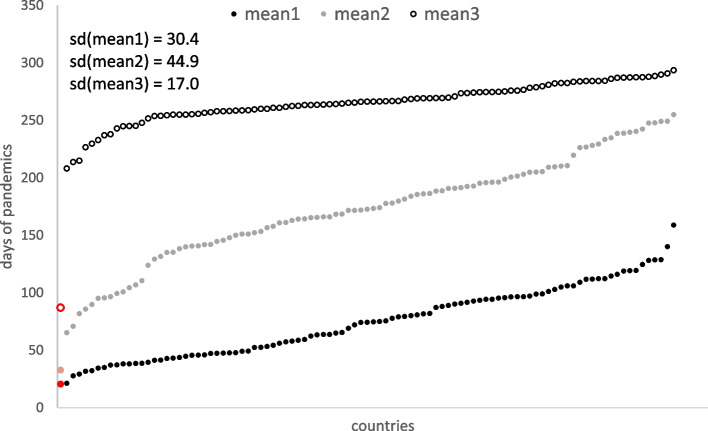
Estimated means of peaks of the number of daily deaths estimated by the linear mixture model for the first 300 days of the pandemic. Means corresponding to China, which was classified as an outlier, are marked in red. Standard deviations were calculated excluding China

Considering the 99 countries which experienced at least 1000 deaths assigned to the SARS-CoV-2 infection during the first 300 days of the pandemic, we observed that the earliest peaks, expressed by the estimated means of the mixture of three normal distributions were attributable to China (20^th^, 33^rd^, and 87^th^ day of pandemic). China is also the only country classified as an outlier, based on the Local Outlier Factor approach applied to the three estimated means of the fitted normal distributions (Fig. 6). The latest first peak of the number of deaths appeared in Argentina, on day 159, the latest second peak on day 255 occurred in Germany, while the third peak was the latest in Zimbabwe, by day 294. The latest peak in the number of daily deaths showed the least variation in time and each country (excluding China as the outlier) appeared after the 200^th^ day of the pandemic (Fig. 6).

## Discussion

Globally, a much greater variation was observed in recovery rates than in mortality rates. Significant sources of heterogeneity in SARS-CoV-2 associated mortality rates across countries are differences in governmental policies related to social aspects of life during the pandemic [11], country globalization level [12], and genetic differences between country- and continent-specific populations [13]. Some of our findings reflect the abovementioned sources of inter-country variation. Australia which was outlying in the numbers of confirmed cases over time by showing two, rather than three peaks over the first 300 days of the pandemic, and China outlying by having all three peaks of confirmed deaths estimated in a short time-span one to another were previously mentioned by Pearce et al. [14] as countries that imposed early restrictions to social contacts. Moreover, high mortality rates estimated by us using the SIRD model aligned with highly globalised countries (Belgium, France, United Kingdom, Italy, Hungary, and the Netherlands for D150). However, neither the patterns of outlying countries nor the sole estimated mortality rates did align with their geographical locations as it may have been expected providing genetic differences between populations, a phenomenon also observed by Balmford et al. [11]. Moreover, countries classified as “positive” outliers with high recovery rates and low mortality rates also did not show any outstanding values of socio-economic factors.

There are several limitations of this study, which we are aware of. Firstly, one must consider the unreliability of the reported numbers. It is widely known that testing rates vary greatly amongst the studied countries. Moreover, it is also believed that some nations, falsify the pandemic statistics or at least underreport cases and deaths with full awareness of it. Secondly, statistical limitations arise from the nature of the SIRD model itself. The mentioned model has several assumptions that are not fully adequate for the COVID-19 pandemic. One of them is the assumption of random mixing, which implies that any two given individuals have the same chance of meeting each other. This of course is not true in a real-world scenario, where commonly people tend to have more contact within their age and social groups. The SIRD model also does not take the effect of vaccinations, maternally-derived immunity, or the waning of immunity into account. These are just some examples of why the SIRD model might be an oversimplification of the pandemic dynamics. However, considering the aforementioned problems with data reporting quality and its variation among countries, we still believe that the application of a robust and simple model, like SIRD, may have modelling advantages over more sophisticated models that involve the estimation of a large number of parameters.

Further lines of research could naturally incorporate the inclusion of more countries, which would add more validity to our findings. It would also be of great value to investigate the pandemic statistics that stem from later periods. We could then possibly see the effect of vaccinations or actions imposed by countries to limit the spread of the pandemic. Moreover, taking further factors, in the SIRD model, into account could be an option. For instance, adding a WAIF (who acquires infection from whom) matrix into our study.

## Conclusions

Considering both major outcomes of the SARS-CoV-2 pandemic, i.e. the recovery and death, heterogeneity between countries exists. The goal is to be classified as an outlying country based on exceptionally low mortality rates and high recovery rates. Liechtenstein was the closest to this favourable pattern being a “positive” outlier for D30, D60, and D150. On the other end, Yemen represents a “negative” outlier with high mortality rates for all four considered periods and low recovery rates for D30 and D60. We believe that the observed country-specific differences result from a mixture of factors including the biology of the virus strains, different policies adopted by countries to mitigate the spread of infections, but also different accuracy of data reporting.

## Data Availability

The input data underlying this article is publicly available from the SARS-CoV-2 Data Repository at https://github.com/CSSEGISandData/COVID-19.
